# Myth Dispelled

**DOI:** 10.3201/eid1904.AD-1904

**Published:** 2013-04

**Authors:** Adam Possner

**Affiliations:** George Washington University, Washington, DC, USA

**Keywords:** influenza, viruses, vaccine

The flu vaccine cannot

give you the flu, I tell him.

It’s dead virus, there’s

nothing alive about it.

It can’t make you sick.

That’s a myth.

But if we bury it in

the grassy knoll

of your shoulder,

an inch under the stratum

corneum, as sanctioned by

your signature

in a white-coated ceremony

presided over by

my medical assistant

and then mark the grave

with a temporary

non-stick headstone,

the trivalent spirit

of that vaccine

has a 70 to 90 percent

chance of warding off

the Evil One,

and that’s the God’s

honest truth.

